# Enhanced spectral resolution and reduced acquisition time in fiber-based wavelength-swept source Raman spectroscopy

**DOI:** 10.1117/1.NPh.12.1.015014

**Published:** 2025-03-13

**Authors:** Elahe Parham, Maxime Tousignant-Tremblay, Mireille Quémener, Martin Parent, Daniel C. Côté

**Affiliations:** a CERVO Brain Research Center, Québec, Canada; b Université Laval, Centre d’Optique, Photonique et Laser, Québec, Canada

**Keywords:** Raman spectroscopy, wavelength-swept, fast Raman spectroscopy, tissue identification, signal processing

## Abstract

**Significance:**

We introduce a fast Raman spectroscopy (SSRS) system that reduces acquisition time and enhances data quality, providing a breakthrough in SSRS for real-time applications. We demonstrate its utility in differentiating brain tissue regions based on lipid and protein content.

**Aim:**

Our primary goal was to develop a fast SSRS system that enables rapid data acquisition for *in vivo* applications. We aimed to investigate its effectiveness in differentiating brain tissue types by analyzing lipid and protein content, ultimately enhancing classification accuracy and supporting advancements in medical diagnostics.

**Approach:**

We implemented an optimized circuit and signal processing technique to reduce high-frequency noise and improve signal-to-noise ratio. Brain tissue measurements were validated against staining models, and classification accuracy was tested with principal component analysis (PCA) and support vector machine (SVM).

**Results:**

Our SSRS system captures spectra in 1 s which is significantly faster than similar systems. This rapid method enables real-time monitoring and accurate classification of brain regions based on lipid–protein content, confirmed by neurofilament and Nissl staining correlations ($R^{2}=0.75$ and 0.55, respectively). Tissue classification showed 80.20% accuracy using spectral intensity at the wavenumbers associated with C–H, ${\mathrm{CH}}_{3}$, and ${\mathrm{CH}}_{2}$ vibrations and 81.23% accuracy using PCA-derived features (PC1, PC2, and PC3).

**Conclusions:**

The fast-SSRS system marks a significant advance in Raman spectroscopy, improving speed and data quality. Our setup captures finer spectral details, facilitating reliable differentiation of tissue types, as verified by staining methods and PCA. This method shows promise for real-time tissue analysis and medical diagnostics, outperforming traditional Raman techniques in speed and data throughput.

## 1 Introduction

Raman spectroscopy provides detailed information about the vibrational modes of molecules by measuring the inelastic scattering of photons and enables the identification and quantification of chemical species with high specificity.^1^ It has been a powerful analytical technique for the non-invasive and label-free characterization of molecular structures in biomedical research for decades^2^^–^^4^ and has found applications both in the laboratory^1^^,^^5^^,^^6^ and in the clinic.^7^^,^^8^ Raman microscopy has shown promising results for analyzing Alzheimer’s disease brain tissue, providing fast, label-free, high-resolution imaging of molecular changes.^9^ It is typically performed with a dispersive spectrometer and suffers from relatively low signals due to the tight constraints on the input beam size, which becomes particularly critical in highly scattering tissues.

For this reason, Swept-source Raman spectroscopy (SSRS) has been introduced recently.^10^ In this method, a tunable laser source is used to excite the sample, and a sensitive photodetector with a narrow bandpass filter is employed in combination with a continuous sweep of the wavelength to acquire the spectrum over the entire range, one wavenumber at a time.

Unlike conventional Raman spectroscopy, which relies on dispersive spectrometers, using a photodetector helps to overcome the limitation of low photon collection and detection. SSRS has answered the need for portable, low-cost Raman spectrometers that are compatible with low-power lasers. A recent study^11^ showcased that using a swept source laser and a detector, instead of a dispersive spectrometer, reliable Raman spectra from chemical and biological samples can be obtained. Atabaki et al. introduced a sensitive Raman spectroscopy method that uses low excitation power and uncooled detectors. The technique employs miniature chip-scale lasers and low-cost photodiodes to characterize molecular species in everyday items such as painkillers, vegetables, and alcohol. The approach aims to simplify Raman spectroscopy and broaden its accessibility by reducing the size and cost of equipment.^12^ This approach could also be employed to detect low-concentration chemicals with Raman spectroscopy.^13^

Our recent research^10^ introduced a fiber-based swept source Raman spectrometer. We have shown that for a similar illumination power, SSRS collects at least 200× more photons compared with spectrometer-based Raman spectroscopy thanks to the larger area and acceptance cone of the detector. Employing a detection system with a substantially expanded *optical invariant* enables the system to capture and harness the scattered photons from a beam with a large* étendue*, avoiding losses from scattering effects within biological tissue and thus enhancing the reliability and accuracy of the measurements. In addition, the fiber-based SSRS system also shows promise for *in vivo* tissue identification, particularly in highly scattering media such as the brain.

Despite all the advantages, the system previously introduced has a speed constraint that limits the possibility of using it *in vivo*; otherwise, it would heat up the tissue. In addition, it uses a lock-in amplifier for narrowband detection without requiring rapid motor movement. The detected signal is at near-zero frequency which is less desirable due to low-frequency noise, known as 1/f noise, that increases as the frequency decreases.^14^ To minimize this noise, we aim to avoid static or low-frequency measurements by continuously moving the motor and focusing on broadband acquisitions at higher frequencies.

The primary goal of our study is to extract and analyze vibrational information from various brain regions. We believe this information will reveal important differences, such as the presence of specific neurotransmitters, which are crucial for understanding the brain’s biochemical composition. Detailed vibrational data could significantly enhance the precision of intraoperative brain mapping, enabling surgeons to accurately delineate and target specific regions based on their biochemical signatures.^15^^,^^16^ This capability could lead to more effective and targeted interventions, minimizing the risk of damage to adjacent tissues and improving clinical outcomes. Our approach aims to facilitate brain region identification and precise surgical navigation, ultimately contributing to advancements in neurosurgery and related fields.

In this study, we dramatically enhanced the system by replacing the narrowband detection of the lock-in amplifier with rapid scanning and fast digitization with a Moku:Go benefiting from the very high analog-to-digital converter (ADC) resolution and the high sampling rate of Moku. We also implemented electronic filters and amplifiers for more effective signal management to move away from DC detection. We demonstrate the application of the newly developed SSRS setup in studying brain tissue and performing hyperspectral imaging with a gain in speed of 45× compared with our previous study.

## 2 Method and Material

### 2.1 Experimental Setup

The experimental SSRS setup introduced in this paper uses the same components as the introduced setup in our recent study.^10^ The swept-source Raman Spectrometer (SSRS) is a Ti:Sapphire MIRA 900 (Coherent) pumped by a 532 nm Verdi G (Coherent) laser. The Mira configuration has been modified to operate the laser in continuous wave (CW) in the auxiliary cavity without dispersion compensation. In addition, the screw knob of the birefringent filter (0163-800-50, Coherent) inside the laser has been replaced with a stepper motor actuator (ZST206, Thorlabs) connected to a k-cube stepper motor controller (KST101, Thorlabs) to automatically tune the excitation wavelengths from 800 to 820 nm. The birefringent filter has been replaced by the low bandwidth model (Coherent, 3-PLATE BRF Ti:S). The average bandwidth of the excitation wavelength is 1.5 nm. A bifurcated fiber bundle (BFY600HS02, Thorlabs) with a 600-μm core and an NA of 0.39 is used to deliver the laser light (150 mW at the sample) and collect the scattered light from the sample, which is positioned approximately 1 mm directly in front of the probe. An ultra-narrow bandpass filter 1064/1 nm (Alluxa) and a low-bandwidth (750 Hz) InGaAs femtowatt photoreceiver (Model 2153, Newport) are employed for signal collection, covering the Raman shift region from 2800 to $3100\;{\mathrm{cm}}^{-1}$. This range provides valuable information about the molecular vibration of lipids, ${\mathrm{CH}}_{2}$, and ${\mathrm{CH}}_{3}$ bonds, offering insight into the composition of the cell membrane and myelin in the brain. It is worth mentioning that utilizing the narrowband filter in front of a large area detector with a large input NA will support a much larger invariant if the beam is expanded to fill the filter to maintain its narrow linewidth and, hence, will detect a beam with a much larger *étendue*.

**Fig. 1:**
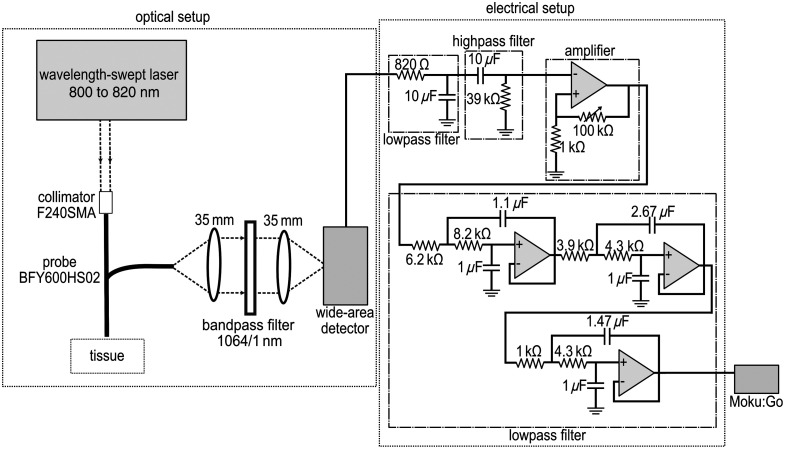
Schematic of the improved wavelength-swept source setup. The swept source is a Ti:Sapphire MIRA 900 (Coherent). The laser is injected into the probe (BFY600HS02, Thorlabs) with a collimator (F240SMA, Thorlabs). The collected light passes through a 4f system (two lenses with a focal length of 35 mm) to expand the beam and reduce its divergence so that an ultra-narrow bandpass filter 1064/1 nm (Alluxa) can be used before it reaches a femtowatt photoreceiver (Model 2153, Newport). The output of the detector goes through a 1st LP filter (19 Hz), then an HP filter (0.4 Hz), and then amplified. Finally, after going through a 2nd LP filter (cut-off frequency: 18 Hz), it is acquired with Moku:Go.

The photoreceiver is connected to an RC low pass (LP) filter at 19 Hz and then an RC high pass (HP) filter at 0.4 Hz. After reducing the noise, it goes through a non-inverting amplifier (LMC648 operational amplifier) with a maximum gain of ∼1000. The output of this step is filtered again with three second-order Sallen-Key filters to obtain a smooth and less noisy signal (cut-off frequency: 18 Hz, shown in Fig. 1). The output is then connected to the input of the Moku:Go. The second input channel of Moku:Go is connected directly to the output trigger of the motor controller on the laser. So when the motor starts the movement, i.e., changing the excitation wavelength, the output trigger of the motor is High (5 V), and using the data streaming function of the Moku:Go, all the data during the movement of the motor (1 s) is acquired. A homemade program in Python was employed to manage the stepper motor movement (wavelength tuning) and the acquisition from the Moku:Go. The setup is shown in Fig. 1.

In the previous paper,^10^ i.e., the setup with the lock-in amplifier, there is a chopper right after the laser operating at 320 Hz. In addition, instead of the electrical setup in Fig. 1, the wide-area detector is connected directly to the lock-in amplifier (SR830, Stanford Research System) to enable phase-locked detection with a time constant of 300 ms. To simplify, we will refer to the setup utilizing the lock-in amplifier as “slow-SSRS” and the alternative setup using Moku:Go as “fast-SSRS.”

### 2.2 Brain Tissue Preparation

Experiments involving the post-mortem monkey brain were approved by the Comité de Protection des Animaux de l’Université Laval, in accordance with the Canadian Council on Animal Care’s guide to the Care and Use of Experimental Animals. The brain is from a cynomolgus monkey (*Macaca fascicularis*) aged 4 years old and weighing 2.9 kg. The monkey was sacrificed by an overdose of sodium pentobarbital, and the whole brain was extracted and fixed by immersion in paraformaldehyde 4% for 4 days. Transverse brain sections of 1 mm were obtained using vibratome and collected serially in phosphate-buffered saline (PBS, 0.1 M, pH 7.4).

### 2.3 Mapping Brain Regions Using Raman Spectra

Using 1-mm-thick brain slices, we acquired 116 spectra, 26 from WM, 10 from the subthalamic nucleus (STN), 24 from globus pallidus (GP), 10 from substantia nigra (SN), and striatum [31 from putamen (Put) and 15 from the caudate nucleus (Cd)]. Each spectrum is obtained from the average of 50 acquisitions at a single point. The brain regions were identified by a neuroanatomist using a stereotaxic atlas of *Macaca fascicularis* brain.^17^

### 2.4 Hyperspectral Imaging of the Brain Tissue

We have used our fast-SSRS system for hyperspectral imaging. To add this feature to the setup, we employed a controller and micromanipulator (MPC-220 and ROE, Sutter instrument) to move the sample automatically along the x and y-axes. The sample is placed on a quartz microscope slide. Using a lens and a camera, we obtain images from the bottom view. At the same time, the fiber is in a fixed place on the top of the sample. The bottom part including the sample, sample holder, lens, and camera (CS165MU, Thorlabs) are attached to the 3D stage controlled by the micromanipulator. Using a brain slice with several regions of interest (putamen, caudate, external globus pallidus (GPe), internal globus pallidus (GPi), substantia innominata (SI), and three areas with WM including internal capsule (ic), external capsule (ec), and optic tract (ot)), we have scanned a 12  mm×8  mm area with 1 mm distances between each measurement. Although the spatial resolution is not high, it is enough to differentiate structures in the brain tissue.

## 3 Result

**Fig. 2:**
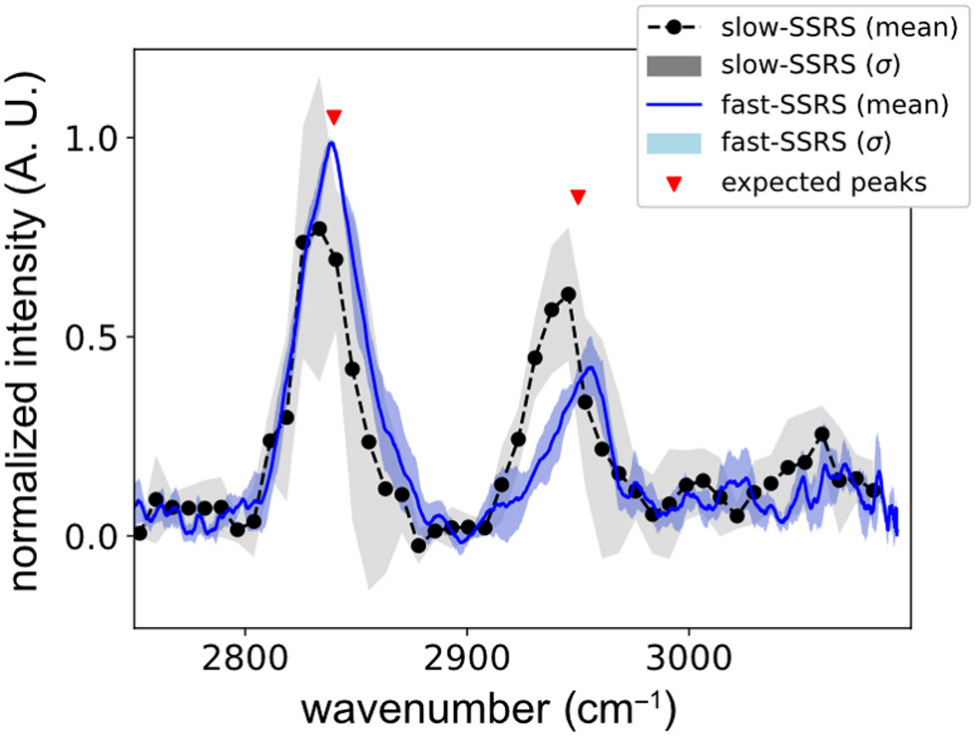
Average and standard deviation of Raman spectra of methanol were obtained with slow-SSRS and fast-SSRS for five acquisitions. The total acquisition time for obtaining five spectra with slow-SSRS is 225 s versus 5 s for fast-SSRS.

Methanol is an organic chemical compound with a well-known Raman spectrum. The spectrum of methanol in high wavenumbers is expected to have two peaks around 2840 and $2950\;{\mathrm{cm}}^{-1}$, generated by CH3 symmetrical and asymmetrical stretching vibrations.^18^ The average Raman spectra obtained from five acquisitions of methanol with the slow- and fast-SSRS setups are plotted in Fig. 2. The baseline of the two spectra is removed with the bubble-fill baseline removal algorithm.^19^

In Fig. 2, the spectrum obtained with slow-SSRS includes 45 data points, i.e., 45 different wavelengths, and each point takes 1 s. On the other hand, the spectrum obtained with fast-SSRS is recorded in a single movement of the motor (one sweep of all wavelengths) that takes 1 s with a sampling rate of $10^{4}\;\mathrm{Hz}$. Therefore, the spectrum with fast SSRS is 45× faster than the slow SSRS.

**Fig. 3:**
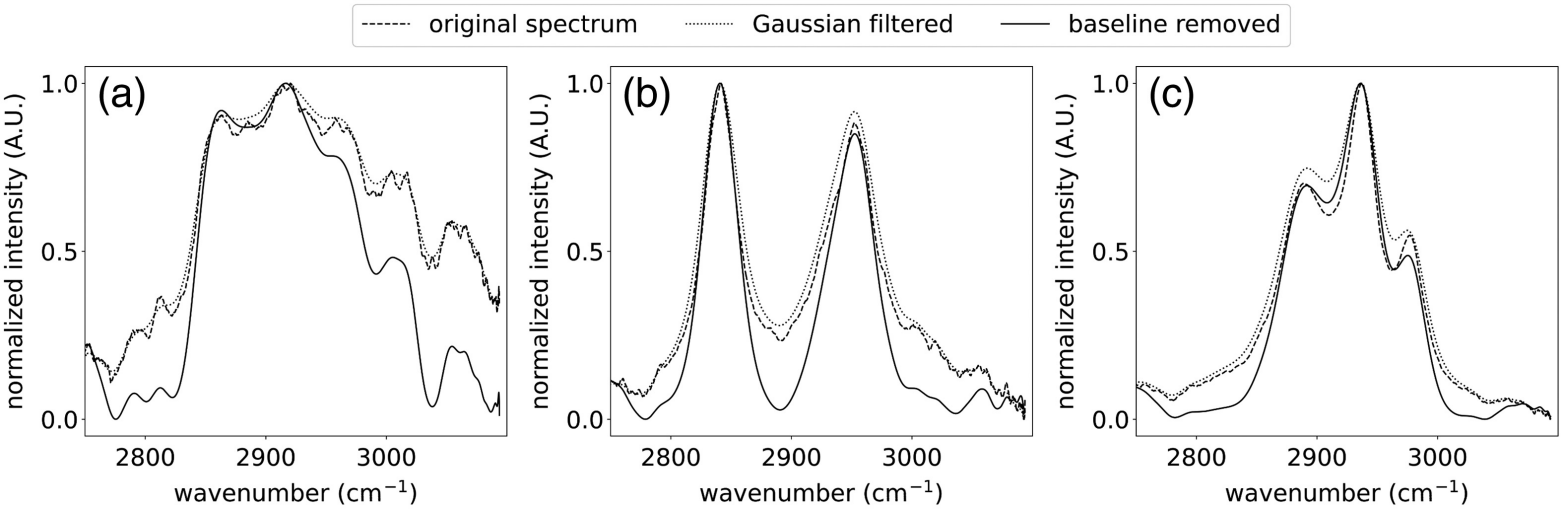
Original Raman spectra were obtained from (a) brain WM, (b) methanol, and (c) ethanol. The original signal is then filtered using a Gaussian with a standard deviation of 20 pixels to smooth the spectrum. Finally, the baseline is removed with the bubble-fill algorithm with a size $200\;{\mathrm{cm}}^{-1}$.

For the post-processing steps, we have downsampled the signals to 5000 points. Then, we use a Gaussian window to smooth the variation in the signal. The standard deviation of the Gaussian is set to 20. The selected standard deviation should not be large because it can broaden the Raman peaks in the signal while it filters out the noise. The processing steps are applied to spectra obtained from three different samples including brain white matter (WM) [Fig. 3(a)], methanol [Fig. 3(b)], and ethanol [Fig. 3(c)]. Although the spectrum obtained with fast-SSRS can be improved with signal processing techniques, the resolution of the spectrum is limited by system constraints, i.e., the bandwidth of the laser (∼1.5  nm) and the bandpass filter (1 nm).

**Fig. 4:**
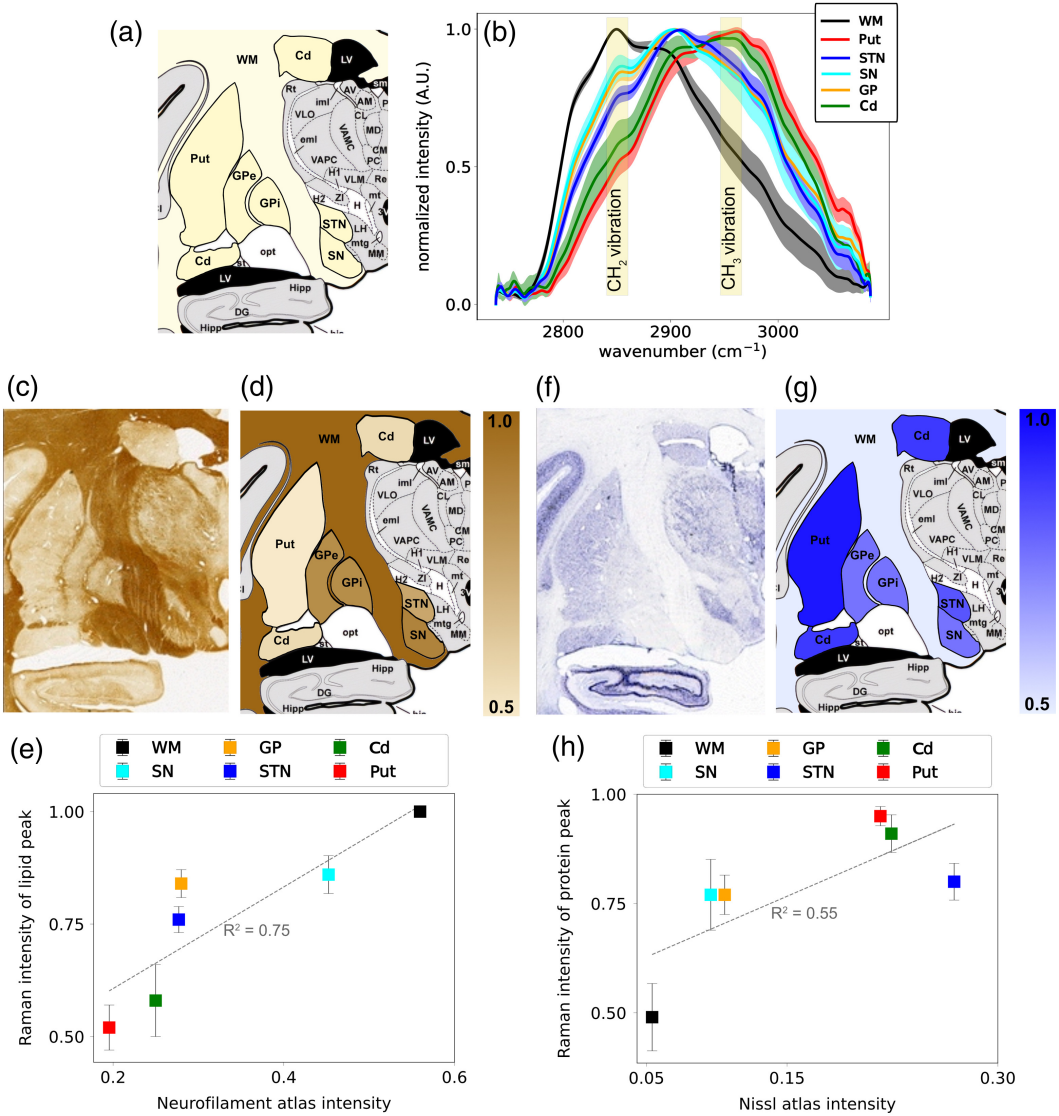
(a) Targeted regions to obtain spectra are highlighted in yellow. (b) The average and standard deviation of the normalized spectra obtained from each region. (c) Coronal section (anterior commissure −5.5  mm) of the Neurofilament atlas.^21^ (d) The average intensity of the signal at $2850\;{\mathrm{cm}}^{-1}$ is used to color the atlas (associated with the concentration of lipids^20^). (e) The scatter plot of the normalized intensity of the lipid peak and the average intensity of the region on the neurofilament staining image. (f) Coronal section (anterior commissure −5.5  mm) of the Nissl atlas.^21^ (g) The average intensity of the signal at $2965\;{\mathrm{cm}}^{-1}$ is used to color the atlas (associated with the concentration of proteins^20^). (h) The scatter plot of the normalized intensity of the protein peak and the average intensity of the region on the Nissl staining image.

We have targeted several brain regions including WM (ic, ec, and ot), putamen (Put), caudate nucleus (Cd), globus pallidus (GP), substantia nigra (SN), and subthalamic nucleus (STN). They are highlighted in yellow on a coronal brain section (anterior commissure −5.5  mm) shown in Fig. 4(a). This section includes all relevant basal ganglia components and thalamic nuclei of interest for this study. The average and standard deviation of the obtained spectra from each region are plotted in Fig. 4(b). We can also see the intensity of the signal at $2850\;{\mathrm{cm}}^{-1}$ (associated with the concentration of lipids^20^) and $2965\;{\mathrm{cm}}^{-1}$ (associated with the concentration of proteins^20^). Figure 4(c) depicts a transverse brain section from a cynomolgus monkey taken from the atlas that was immunostained for neurofilament and Fig. 4(f) is the same section from the Nissl-stained atlas.^21^ The intensity observed in the neurofilament method is proportional to the optical density of lipids. By contrast, the Nissl method, which binds to RNA and ribosomes and stains the rough endoplasmic reticulum, is expected to produce an inverse appearance compared with the neurofilament-stained sections. Typically, WM appears white in the Nissl staining but dark in the neurofilament staining method. Figure 4(d) shows the average intensity of the spectra at $2850\;{\mathrm{cm}}^{-1}$, which is associated with the lipid content of the brain tissue (${\mathrm{CH}}_{2}$ vibration), for the regions of interest. In addition, the normalized average intensity value of the regions versus the average intensity of each region in Fig. 4(c) is plotted in Fig. 4(e). The lipid in tissue mostly comes from the myelinated axons in white matter. Because Fig. 4(c) shows a staining for neurofilaments that are largely contained in axons often surrounded by myelin, it is comparable with Fig. 4(d). Figure 4(g) is the intensity of the spectra at $2965\;{\mathrm{cm}}^{-1}$, which is associated with the concentration of proteins (${\mathrm{CH}}_{3}$ vibration). We can compare Fig. 4(g) with Fig. 4(f) and observe the same trend in the intensities. There is generally more protein content in cell bodies compared with the myelinated axons in WM. The normalized average intensity value of the regions in Figs. 4(g) and 4(f) is plotted in Fig. 4(h).

**Fig. 5:**
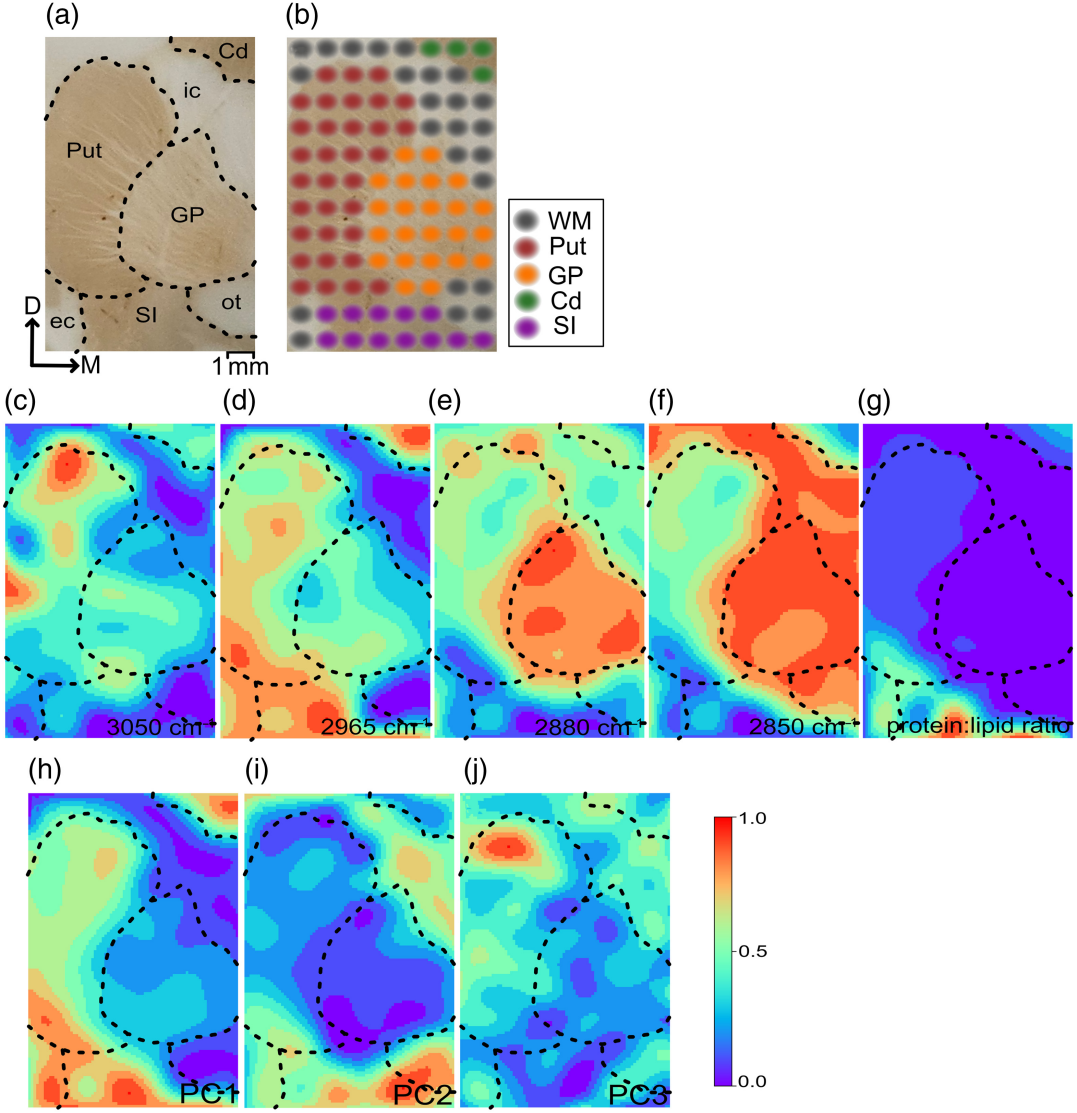
(a) The brain slice used for the hyperspectral imaging including putamen (Put), caudate nucleus (Cd), globus pallidus (GP), substantia innominata (SI), and WM (including internal capsule (ic), external capsule (ec), and optic tract (ot)). The regions are separated by dashed lines. (b) The spots from where the spectra were taken. Each point is colored according to the region that was labeled. (c) The normalized intensity at $3050\;{\mathrm{cm}}^{-1}$ associated with C–H vibrations.^25^ (d) ${\mathrm{CH}}_{3}$ symmetric vibrations at $2965\;{\mathrm{cm}}^{-1}$ associated with protein. e) ${\mathrm{CH}}_{2}$ asymmetric vibrations ($2880\;{\mathrm{cm}}^{-1}$). (f) ${\mathrm{CH}}_{2}$ symmetric vibrations at $2850\;{\mathrm{cm}}^{-1}$ associated with lipids. (g) protein:lipid ratio. The transformed spectra with PCA analysis for (h) PC1, (i) PC2, and (j) PC3.

The brain slice used for hyperspectral imaging is shown in Fig. 5(a), and the regions of interest are separated by dashed lines. Figure 5(b) shows the spots where each spectrum was measured and the spots are colored to show the identified label for each point. The normalized intensities of the obtained spectra at each point for $3050\;{\mathrm{cm}}^{-1}$, $2965\;{\mathrm{cm}}^{-1}$ (protein), $2880\;{\mathrm{cm}}^{-1}$, $2850\;{\mathrm{cm}}^{-1}$ (lipid), and protein:lipid ratios are shown in Figs. 5(c)–5(g). To obtain these figures, we have upsampled the 8×12  pixel images obtained at each wavenumber to 80×120  pixel images using the two-dimensional interpolation function from the Scipy Python package. Then, we rounded the numbers to one decimal place to have clear borders on the plotted images. We represent the intensity of the wavenumbers associated with ${\mathrm{CH}}_{2}$ symmetric vibrations ($2850\;{\mathrm{cm}}^{-1}$),^22^^,^^23^ ${\mathrm{CH}}_{2}$ asymmetric vibrations ($2880\;{\mathrm{cm}}^{-1}$),^23^ ${\mathrm{CH}}_{3}$ asymmetric vibrations ($2965\;{\mathrm{cm}}^{-1}$),^24^ and C–H vibrations ($3050\;{\mathrm{cm}}^{-1}$).^25^

In addition, principal component analysis (PCA) was performed on the spectra. Figures 5(h)–5(j) are the outputs of performing PCA on all of the spectra and repeating the upsampling and thresholding processes for the transformed values associated with the first, second, and third principal components (PC1, PC2, and PC3). The first three PCs account for 87.75%, 13.20%, and 2.19% of the explained variance, respectively. The first PC is positively correlated with protein intensity and negatively correlated with lipid intensity, indicating that the reconstructed image in Fig. 5(h) exhibits higher intensity in regions with elevated protein levels and lower lipid levels. The second PC shows an inverse relationship with the intensity of the ${\mathrm{CH}}_{2}$ asymmetric vibration peak, as depicted in Fig. 5(i). The third PC is associated with protein concentration but not lipid levels, a trend consistent with Fig. 5(j).

**Fig. 6:**
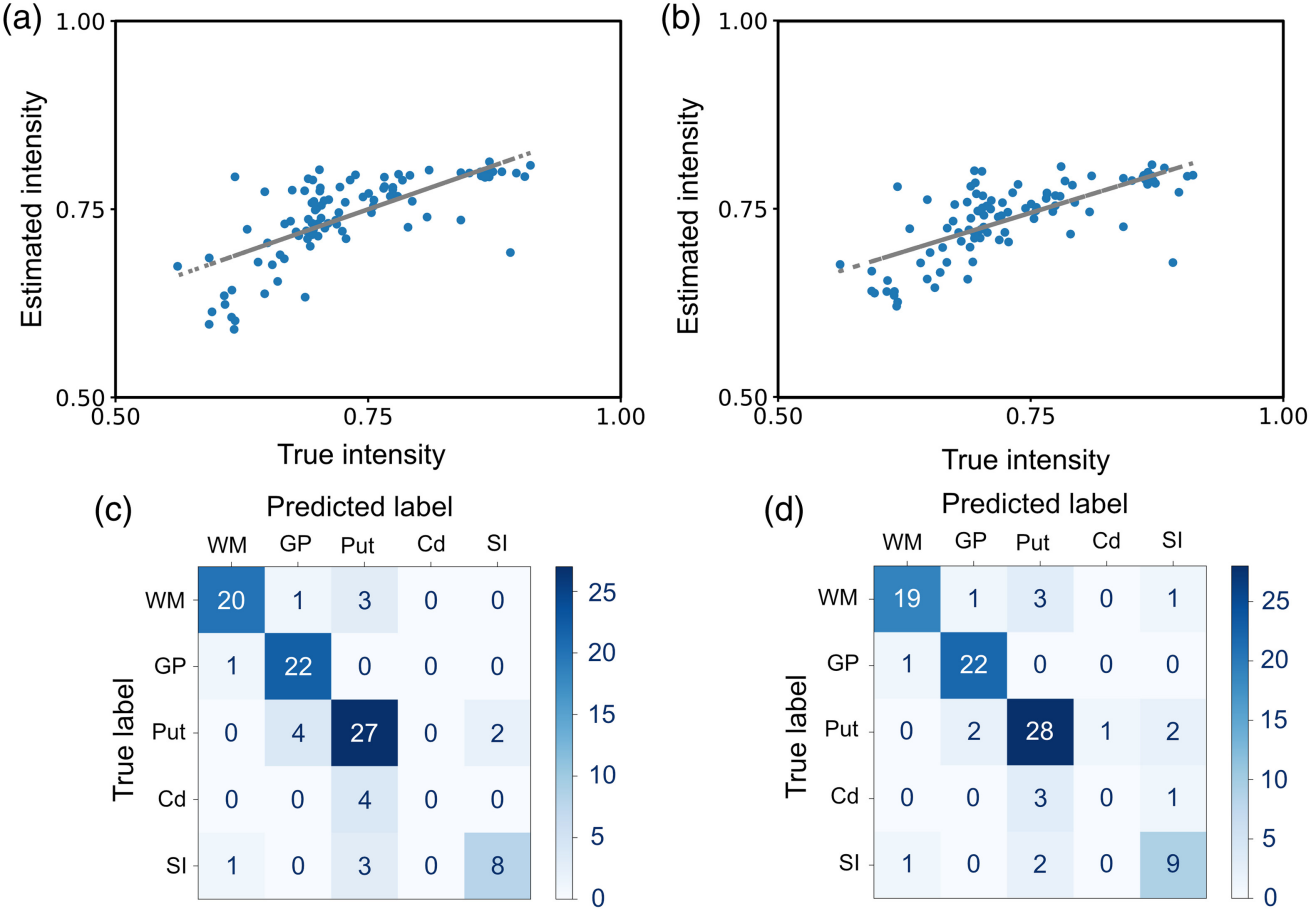
Estimated image intensity using SVR with (a) signal intensities at four selected wavenumbers and the protein:lipid ratio (correlation: 0.70) and (b) the first three principal components, P1, PC2, and PC3 (correlation: 0.70). Classification accuracy using a linear SVC with (c) signal intensities at four selected wavenumbers and the protein:lipid ratio (accuracy: 80.20%) and (d) P1, PC2, and PC3 (accuracy: 81.23%).

We have performed analysis using statistical techniques to better understand the relationship between the selected features (intensity at different wavenumbers and PCs). First, we performed a regression model, support vector regression (SVR), with a leave-one-out approach, to see if the intensity of the brain slice image can be estimated using two different sets of features. To obtain a value for the intensity of the image at each point where spectra were measured, we converted the main image to a grayscale image and downsampled the image by dividing the image into 12×8 squares. Calculating the average of all pixels in each square results in a 12×8 image with the average intensity associated with each spectrum. We performed the regression once with the intensity of the spectra at 3050, 2965, 2880, 2850, and the protein:lipid ratio and another time with PC1, PC2, and PC3. Figure 6(a) shows the estimated and the actual intensities at each point using the selected wavenumbers and their regression line. The Pearson correlation between the two values is 0.70 (p-value: 1.2e−15), and the mean squared error is 3.6e−3. Figure 6(b) shows the estimated and the actual intensities at each point using the PCs and their regression line. The Pearson correlation between the two values is 0.70 (p-value: 1.3e−15), and the mean squared error is 3.8e−3. The observed relationship between the true and estimated values shows a slight deviation from a linear trend. This nonlinearity may arise due to limitations in the estimation model, particularly because the observed intensity on the brain sample may not correlate with the selected feature values, i.e., the intensity of the spectra at the selected wavenumbers or the PC values. Further investigations and validations are needed to confirm this trend.

Second, the same features were used to perform a supervised classification of the regions using the labels shown in Fig. 5(b). Using a support vector classifier (SVC) with a linear kernel and leave-one-out approach, the labels of the regions are predicted and the confusion matrices are shown in Figs. 6(c) and 6(d) for selected wavenumbers (3050, 2965, 2880, $2850\;{\mathrm{cm}}^{-1}$) with protein:lipid ratio and PCs, respectively. The classification accuracy is 80.20% with the selected wavenumbers and 81.23% with the PCs.

## 4 Discussion

Our research group is focused on using fiber-based optical techniques to identify brain tissue during surgery, an area we have made very significant progress over the years.^26^^–^^29^ Using coherent Raman or white light spectroscopy, it has been possible to identify white and gray matter in human brains and identify the insertion tract of an electrode in a simulated deep brain stimulation (DBS) surgery. Yet, it has been difficult to go beyond this identification and harness the power of vibrational spectroscopy to identify more subtle differences, such as the concentration of neurotransmitters. This new wavelength-swept Raman spectroscopy strategy aims to radically enhance the sensitivity and specificity of vibrational spectroscopy under highly constrained conditions, such as high scattering, small optical fibers, and short acquisition times, with the ultimate goal of translating it into clinical use.

In this regard, when comparing total acquisition times, the fast-SSRS operates 45 times faster than the narrowband-SSRS. The narrowband-SSRS system has a fixed acquisition time per point which means the total acquisition time is proportional to the number of points per spectrum. On the other hand, the fast-SSRS has a fixed acquisition time per spectrum, independent of the sampling rate or number of points. The sampling rate can then be adjusted as high as required for the given spectral resolution at no cost.

Overall, the fast-SSRS is also faster compared with similar systems, which require 40 s^11^ and 6 s.^12^ This significant reduction in acquisition time, combined with higher throughput, makes it ideal for real-time monitoring applications.

We developed a signal-processing algorithm to eliminate noise and background interference. A 19 Hz RC low-pass filter was used to minimize high-frequency noise from the detector, while a 0.4 Hz high-pass filter removed the DC component, allowing safe amplification of the Raman signal without saturation. Within the spectral window of 2800 to $3100\;{\mathrm{cm}}^{-1}$, we can observe the peaks corresponding to symmetric and asymmetric ${\mathrm{CH}}_{2}$ and ${\mathrm{CH}}_{3}$, $O-{\mathrm{CH}}_{3}$, and C–H vibrations.^23^^,^^30^ Therefore, a filter with a cut-off frequency higher than 12 Hz is required to detect all relevant peaks. The use of three second-order Sallen-Key filters with a cut-off frequency of 15 Hz effectively minimizes high-frequency noise following amplification.

As previously published,^10^ the fiber-based SSRS setup outperforms the conventional Raman spectroscopy with a dispersive spectrometer in terms of photon collection efficiency in highly scattering media. The preliminary results indicated the method’s potential for accurate classification of distinct brain regions suggesting that SSRS could be the ideal choice for applications involving brain tissue.

The present work, indeed, demonstrates that SSRS spectra obtained from different brain regions can be reliably used to differentiate these regions. Specifically, we focused on the intensity of the Raman spectra at 2850 and $2965\;{\mathrm{cm}}^{-1}$ corresponding to lipids and proteins, respectively.^31^^,^^32^

The signal intensities at wavenumbers associated with lipids (${\mathrm{CH}}_{2}$) and proteins (${\mathrm{CH}}_{3}$) follow patterns similar to those observed with traditional histological staining methods. In Fig. 4, we utilized data from an atlas with different stainings.^21^ The first one labels neurofilaments that are found in high concentrations in axons and consequently in WM, areas composed of myelinated axons. This suggests an interaction between the level of neurofilaments and the amount of lipids form myelin.^33^^,^^34^ The second staining method is the Nissl stain or Cresyl Violet, which labels ribosomal RNA abundant in the cell bodies of neurons.^35^^,^^36^ Because GM is largely composed of neuronal cell bodies and dendrites, these regions stain well for Nissl compared with WM for which only a few neuronal cell bodies are found.^37^ In Figs. 4(d)–4(f), the lipid intensity shows a similar pattern to the neurofilament atlas, with the highest intensity in the internal capsule (WM) and the lowest in the putamen, a GM region with fewer myelinated axons. In Figs. 4(g)–4(i), the WM in the Nissl-stained section exhibits lower intensity due to the absence of neuronal cell bodies, whereas intensity patterns in other areas differ from the Nissl atlas. In addition, we should note that the Nissl staining method highlights the RNA which is an indicator of protein but does not directly label proteins in the tissue.

Hyperspectral imaging enables distinction between brain regions by analyzing multiple wavenumbers, revealing their biochemical composition. Figure 5 shows distinct lipid intensity patterns, with the caudate nucleus having the lowest and the globus pallidus and internal capsule showing the highest. However, the putamen and WM are not visually distinct in lipid content. For protein content, WM has the lowest intensity due to myelinated axons, whereas the putamen and caudate nucleus show the highest protein intensities. Notably, the protein:lipid ratio, commonly employed in stimulated Raman spectroscopy (SRS) to distinguish between tumor and healthy tissue,^38^ serves as a single parameter that effectively differentiates all these regions. Other wavenumbers also provide valuable insights. For example, the intensity of ${\mathrm{CH}}_{2}$ asymmetric vibrations at $2880\;{\mathrm{cm}}^{-1}$, as shown in Fig. 5(e), is particularly high in the GP. One of the possible reasons for this increased intensity could be due to the enkephalin, which is more concentrated in GP compared with the caudate nucleus, putamen, and WM.^39^ Similarly, the C–H vibrations in Fig. 5(c) are predominantly observed in the upper part of the putamen, possibly due to the higher concentration of certain neurotransmitters in that region. It is also shown that chemicals such as homovanillic acid (HVA) and choline acetyltransferase (ChAT) are more concentrated in the upper part of the putamen.^40^ In addition, applying PCA gives insight to enhance the differentiation of brain regions. The first PC highlights areas with high protein and low lipid levels, the second PC relates to ${\mathrm{CH}}_{2}$ vibration intensity, and the third PC is linked to protein concentration.

**Fig. 7:**
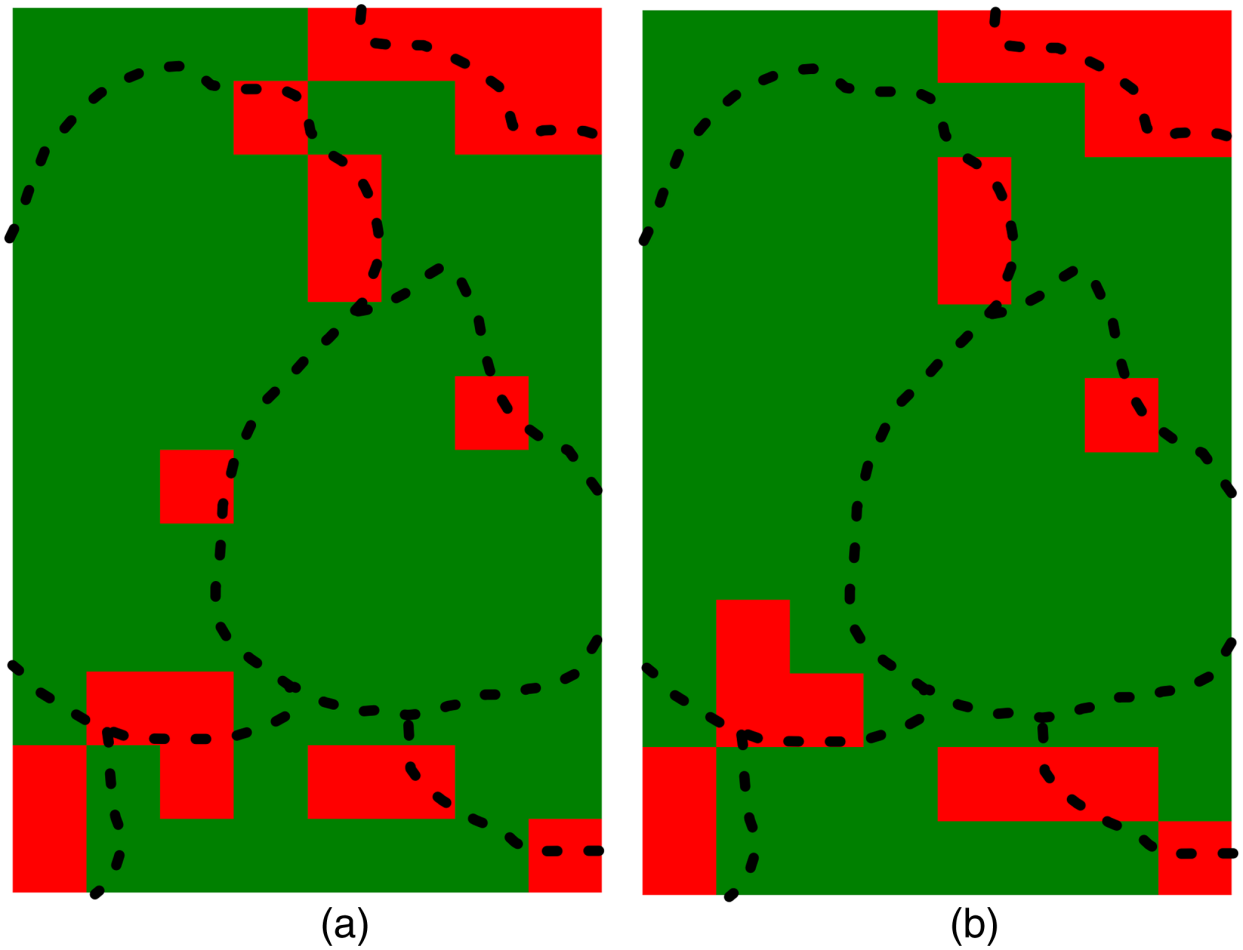
Correctly predicted labels are shown in green, whereas mislabeled regions are shown in red for (a) linear SVC with selected wavenumbers and (b) PC1, PC2, and PC3. The borders of the regions are overlaid on the map.

The accuracy of regression and classification in our study is limited by the system’s spatial resolution. Although the intensity maps in Fig. 5 visually highlight differences in intensity across various regions, the accuracy of the regression and classification is not 100%. This limitation arises because the illuminated area does not always correspond to a single anatomical region. When the measurement point falls between two regions, the results can be compromised, leading to incorrect predictions of intensity or region labels. To further investigate the misclassifications and confirm our hypothesis, we compared the true labels with the predicted labels and plotted the results in Fig. 7. Correctly predicted labels are shown in green, whereas misclassified labels are shown in red. The region borders from Fig. 5 are overlaid on these plots. Most misclassifications occur at the borders between regions. To address this limitation and enhance spatial resolution, a fiber with a smaller diameter and lower numerical aperture can be used for data collection, allowing for a smaller sampling area. However, this approach involves a trade-off between improved spatial resolution and the reduced number of photons collected from the sample. The robustness of classification is also affected by the accuracy of measured spectra, as variability from noise or data acquisition issues can introduce uncertainty in feature values. Improving the signal-to-noise ratio, applying pre-processing (e.g., normalization and removing the baseline), selecting robust features, and using ensemble or robust algorithms can help mitigate these effects.

The current fast-SSRS system is limited by the available hardware. The spectral resolution, which is particularly important for complex materials such as brain tissue, is limited by the bandwidth of both the laser and the narrowband filter. The system can be significantly improved to achieve high spectral resolution, faster operation, and reduced noise with the right equipment. Several sources contribute to the noise in the detected signal. First, although the laser is relatively stable, it is a homemade wavelength-swept source that has limitations in both power stability and sweep speed due to hardware constraints. Replacing it with a more stable, noise-resistant laser capable of faster sweeping would allow us to move further away from low-frequency noise. In addition, the detectors in use, in the NIR range, add high-frequency noise to the signal and can be considered inefficient. Upgrading to a more sensitive photon-counting detector with high efficiency would greatly reduce the noise and result in higher SNR in the obtained spectra. An important consideration for making this system suitable for *in vivo* use is the laser power, which is currently high and may cause a temperature rise in the tissue. Further studies are necessary to determine an optimal excitation power that minimizes the risk of damage to brain tissue.

## 5 Conclusion

The fast-SSRS setup represents a significant advancement in Raman spectroscopy by improving collection efficiency, especially in highly scattering media. The system reduces acquisition time to ∼1  s, a 45-fold improvement over our previous method. This enhancement enables faster data collection, making it suitable for real-time applications such as brain tissue monitoring. Our optimized filtering and signal processing techniques effectively minimize high-frequency noise, resulting in clearer and more reliable spectra.

Spectral analysis revealed distinct lipid and protein intensity patterns across different brain regions, consistent with staining methods. In addition, PCA results highlighted the fast-SSRS setup’s ability to extract meaningful spectral features for tissue classification.

Overall, the fiber-based fast-SSRS setup outperforms conventional Raman techniques in speed and collection efficiency, demonstrating its potential for various applications and paving the way for advancements in medical diagnostics and research.

## Data Availability

Code and data underlying the results presented in this paper are not publicly available at this time but may be obtained from the authors upon request.
